# Extensive Emphysematous Osteomyelitis Attributed to Escherichia coli Infection: Radiological Insights and Detailed Case Report of a Rare Entity

**DOI:** 10.7759/cureus.66912

**Published:** 2024-08-15

**Authors:** Yuvaraj Muralidharan, Sakthi Ganesh Subramonian, Stany Jerosha, Afwaan Faizal, Paarthipan Natarajan

**Affiliations:** 1 Radiodiagnosis, Saveetha Medical College and Hospital, Saveetha Institute of Medical and Technical Sciences (SIMATS) Saveetha University, Chennai, IND

**Keywords:** osteomyelitis, spinal osteomyelitis, escherichia coli infections, emphysematous osteomyelitis, interventional radiology guided drainage, spine imaging, gas forming organism

## Abstract

Emphysematous osteomyelitis (EO) is a rare and severe bone infection characterized by the presence of gas within the bone and surrounding soft tissues, commonly caused by gas-forming bacteria. We present a case of an elderly patient with extensive EO due to *Escherichia coli* infection. The patient exhibited systemic signs of infection and severe localized pain. Radiological assessments, including computed tomography and magnetic resonance imaging, demonstrated significant gas accumulation within the bone and adjacent tissues, confirming the diagnosis. Despite intensive antibiotic treatment and surgical intervention, the patient’s condition initially worsened, highlighting the high morbidity and mortality associated with this infection. However, through prompt action and targeted intervention, a positive outcome was ultimately achieved. This case emphasizes the critical need for early diagnosis and aggressive management of EO to improve patient outcomes.

## Introduction

Emphysematous osteomyelitis (EO) is a rare and potentially fatal infection characterized by the presence of gas within the bone, typically caused by gas-forming bacteria [1]. This condition is most frequently observed in patients with diabetes [2]. Since its first description in 1981 [3], only 49 cases have been documented in the literature, with reported mortality rates reaching up to 44% [2]. Diagnosis is primarily established using computed tomography (CT) scans, which reveal the presence of gas within the bone [4,5]. However, diagnosing EO in the vertebrae can be particularly challenging, as intravertebral gas can also be associated with non-infectious conditions such as degenerative diseases, osteonecrosis, and neoplasms [4-6].

This case report describes a rare instance of widespread EO involving the vertebral bodies, spinal canal, and psoas muscle, caused by an *Escherichia coli* (*E. coli*) infection. The management included surgical debridement and targeted antibiotic therapy, resulting in a successful outcome. This report highlights the critical imaging features of vertebral EO as observed on CT and magnetic resonance imaging (MRI) scans and emphasizes the importance of accurate diagnosis and prompt treatment to mitigate the high risks of morbidity and mortality associated with this aggressive disease.

## Case presentation

A 47-year-old female presented to the emergency department of a tertiary care hospital in South India with symptoms including fever, chills, difficulty walking, low back pain, left hip pain, vomiting, and loose stools for the past week. She also experienced breathlessness for the last three days. Similar symptoms occurred a month prior, treated symptomatically without proper evaluation. Upon presentation, her vital signs were: temperature 100.4°F, pulse rate 89 bpm, and blood pressure 128/84 mmHg, with an initial oxyhemoglobin saturation (SpO2) of 99% on room air. The patient is a known case of type II diabetes and hypertension for 10 years, with no history of tuberculosis or significant surgical history except for sterilization 15 years ago.

Physical examination revealed tenderness over the lower back on palpation and muscle strength of 2/5 in both lower limbs. She reported worsening difficulty walking over the past two days. Initial blood tests indicated neutrophilic leukocytosis (total leukocyte count: 20,870 cells/cu.mm, absolute neutrophil count: 19,190 cells/cu.mm) and anemia (Hb: 8.9 g/dL). The patient had acute kidney injury, as evidenced by elevated renal parameters (urea: 125 mg/dL, creatinine: 2.4 mg/dL) and electrolyte imbalances (serum sodium: 146 meq/L, serum potassium: 3.1 meq/L, serum chloride: 118 meq/L, serum bicarbonate: 13.1 meq/L). Procalcitonin was elevated at 8.94 ng/mL, indicating a high risk for sepsis, and C-reactive protein (CRP) was 83 mg/L. The glycosylated hemoglobin (HbA1c) level was 15.6%. Acid-fast bacillus (AFB) and Mantoux tests were negative (Table 1).

**Table 1:** Lab results with reference range/normal values. HbA1c, glycosylated hemoglobin; AFB, acid-fast bacillus.

Parameter	Result	Reference range	Units
Total leukocyte count (TLC)	20,870	4,000 - 10,000	cells/cu.mm
Absolute neutrophil count (ANC)	19,190	1,500 - 8,000	cells/cu.mm
Hemoglobin (Hb)	8.9	13.8 - 17.2 (Male)	g/dL
		12.1 - 15.1 (Female)	g/dL
Urea	125	7 - 20	mg/dL
Creatinine	2.4	0.6 - 1.2	mg/dL
Serum sodium	146	136 - 145	meq/L
Serum potassium	3.1	3.5 - 5.0	meq/L
Serum chloride	118	98 - 106	meq/L
Serum bicarbonate	13.1	22 - 29	meq/L
Procalcitonin	8.94	< 0.5	ng/mL
C-reactive protein (CRP)	83	< 10	mg/L
HbA1c	15.6	4.0 - 5.6	%
AFB test	Negative	Negative	-
Mantoux test	Negative	Negative	-

**Figure 1:**
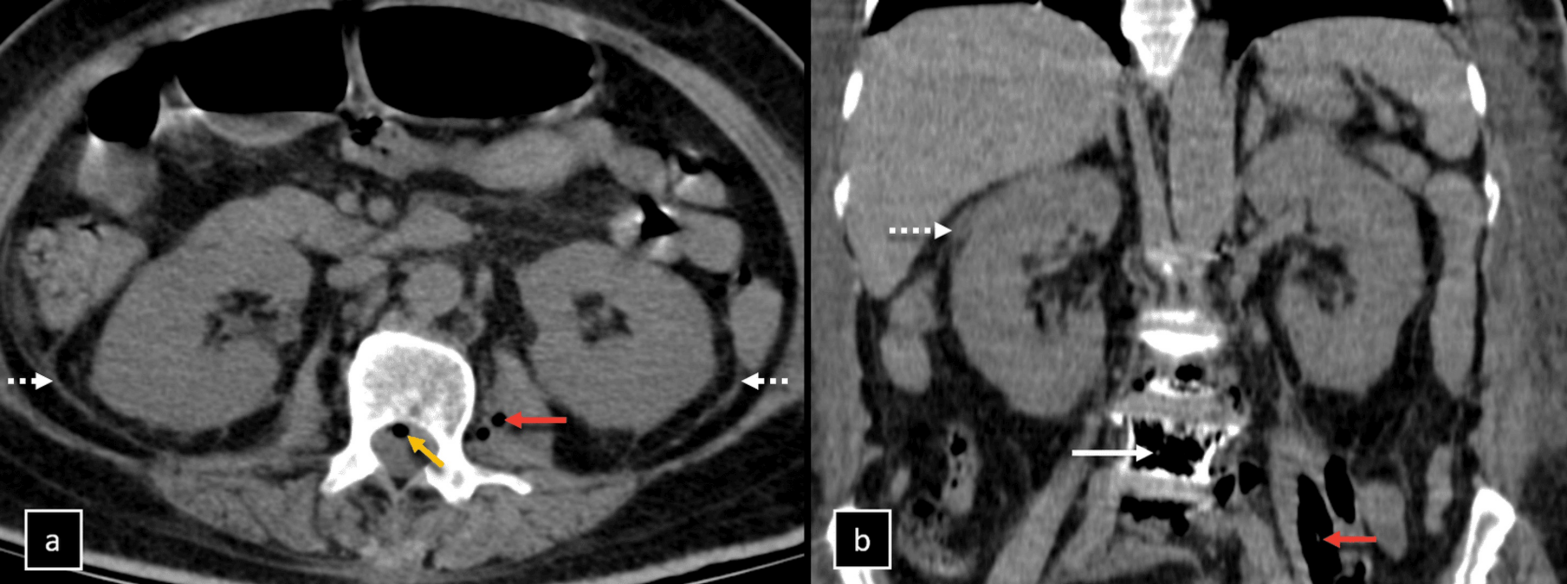
Non-contrast enhanced computed tomography (CT) of the abdomen. Non-contrast enhanced CT of the abdomen, (a) axial and (b) coronal sections, shows a relative globular outline of bilateral kidneys with thickening of bilateral pararenal fascias, peri-nephric and peri-ureteric fat stranding (dashed white arrows). Presence of air within the vertebra (white arrow), left psoas major muscle (red arrow), and spinal canal (orange arrow).

**Figure 2:**
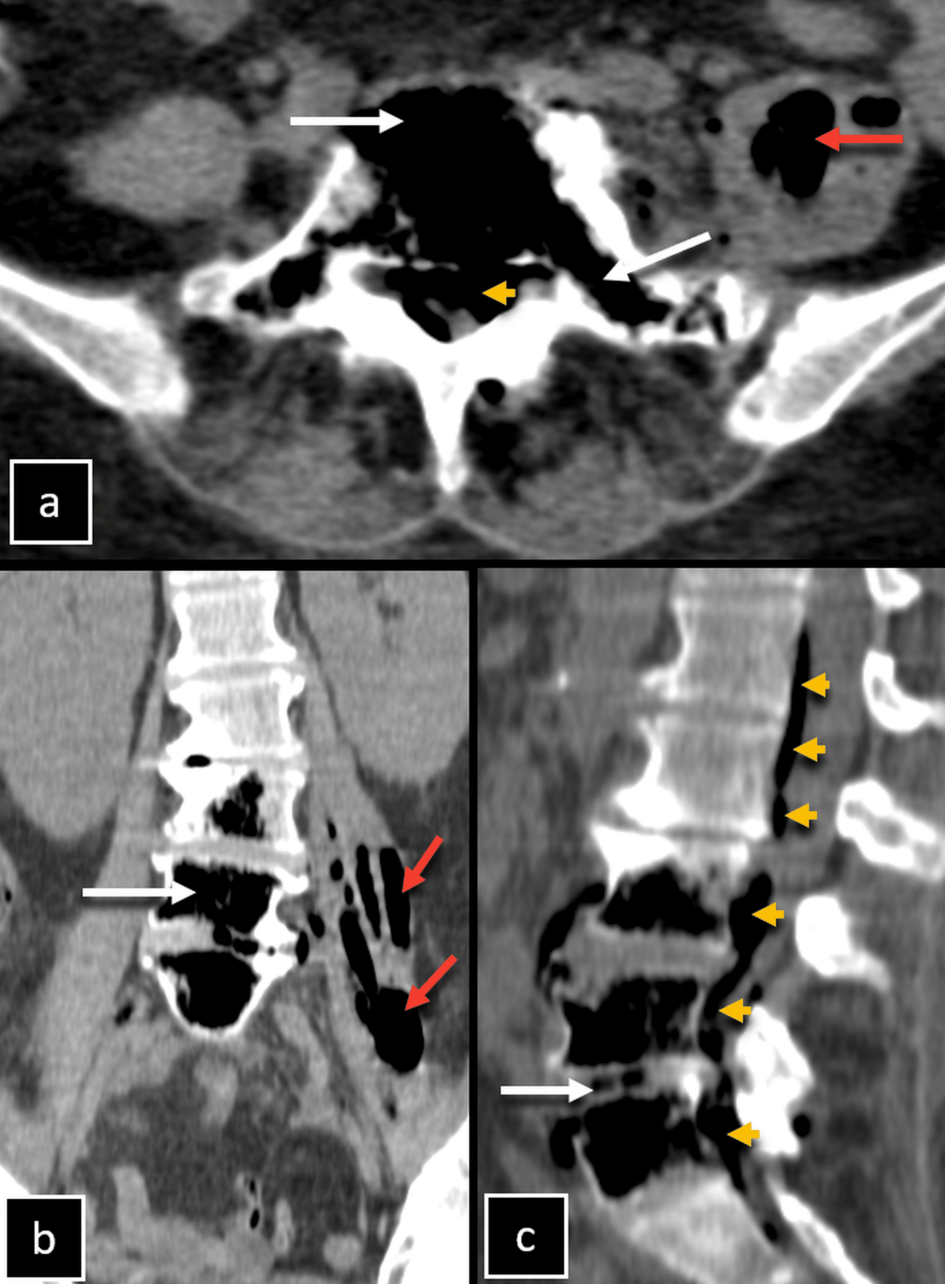
Non-contrast enhanced computed tomography (CT) of the abdomen. Non-contrast enhanced CT of the abdomen, (a) axial, (b) coronal, and (c) sagittal reformatted images show the presence of air within the vertebral bodies of L3 to L5, extending into the transverse processes and pedicle of the L5 vertebra, as well as the intervertebral discs of L2-L3 and L4-L5 (white arrows). The left psoas muscle appears bulky and heterogeneous, with multiple internal air foci (red arrow), and within the spinal canal from L1-S1 level (orange arrowheads).

As she presented with signs of septic shock, further investigations, including a non-contrast-enhanced CT of the abdomen and a chest radiograph, were ordered. The chest X-ray was unremarkable, but the CT abdomen revealed bilaterally bulky kidneys with mild perinephric and periureteric fat stranding and pararenal fascia thickening. There was diffuse intravertebral air from L3 to L5 vertebrae, with air extending into the transverse processes and pedicle of L5, intervertebral discs of L2-L3 and L4-L5, and the extradural space from L1-S1. The left psoas muscle was bulky and heterogeneous, with multiple air pockets (Figures 1, 2).

**Figure 3:**
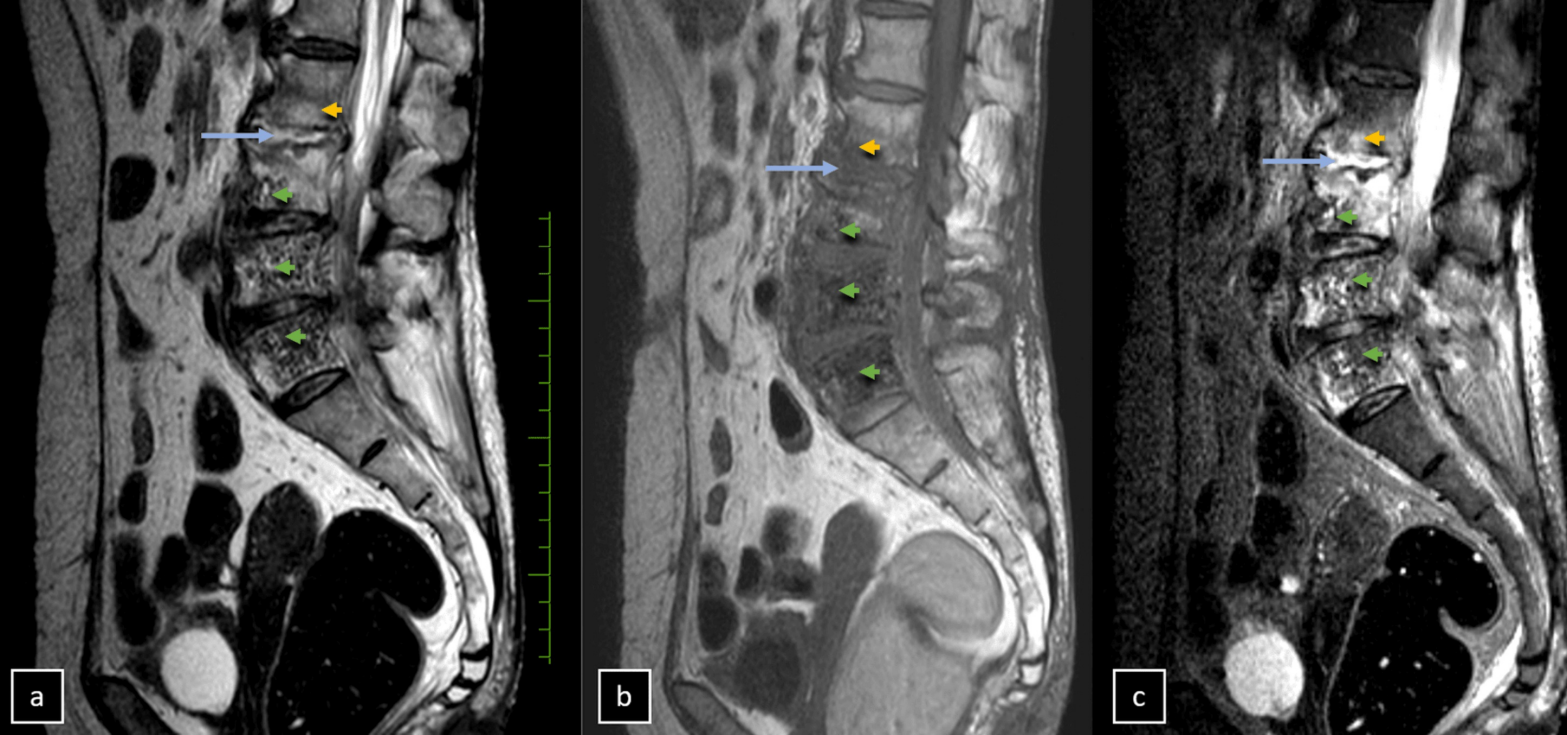
Magnetic resonance imaging (MRI). Sagittal sections (a, b, and c) of T1, T2, and short tau inversion recovery (STIR) sequences depict altered signal intensities in the body of L3, L4, and L5 vertebrae (green arrowheads), suggesting the presence of intra-vertebral air. On T1-weighted images, there is a low signal in the disc space (blue arrow) indicative of fluid, and in adjacent endplates (orange arrowheads), suggesting bone marrow edema. Conversely, T2-weighted and STIR sequences show high signals in the disc space (blue arrow), reflective of fluid accumulation, and in adjacent endplates (orange arrowheads), further indicating bone marrow edema.

The diagnosis of spinal EO with surrounding tissue involvement was made. Given the severity of symptoms, an MRI of the lumbar spine with contrast was performed. The MRI showed altered signal intensities in the bodies of the L3, L4, and L5 vertebrae, appearing heterogeneously hypointense on T1-weighted, T2-weighted, and short tau inversion recovery (STIR) sequences, with no end-plate destruction or disc height reduction observed (Figure 3).

**Figure 4:**
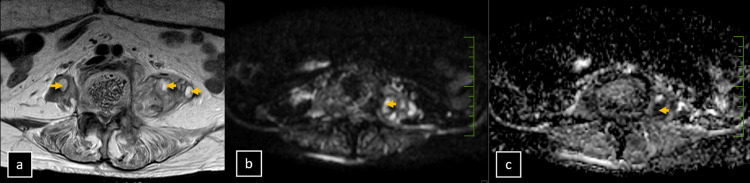
Magnetic resonance imaging (MRI). Axial MRI sections (a, b, and c) including T2-weighted, diffusion-weighted imaging, and apparent diffusion coefficient (ADC) maps illustrate a psoas abscess with concurrent inflammatory signs. Hyperintensity within the psoas muscle is evident in the (a) T2-weighted image, diffusion restriction is shown in the (b) diffusion-weighted image, and (c) the ADC map exhibits corresponding low ADC values (yellow arrowheads).

**Figure 5:**
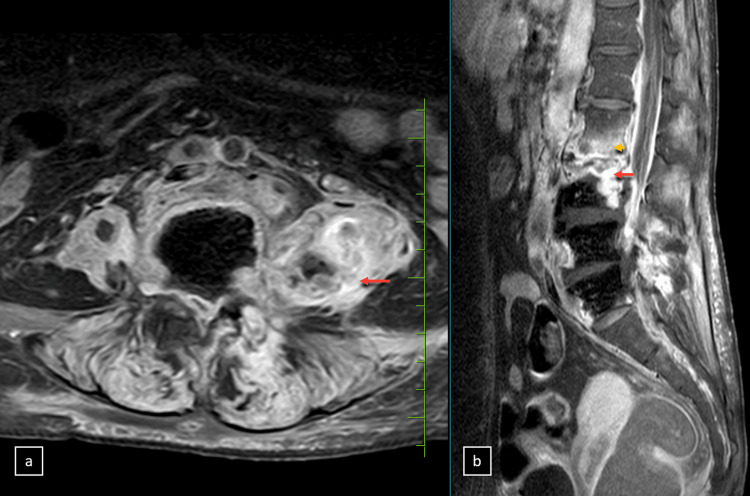
Magnetic resonance imaging (MRI). (a) Axial (b) and sagittal sections of post-contrast images show peripheral enhancement around fluid collections, enhancement of paravertebral soft tissues (red arrows), and enhancement of vertebral endplates (orange arrowheads).

An associated epidural component from L1 to L5 caused significant spinal cord narrowing and severe compression of traversing nerve roots, particularly on the left. Signal voids within the epidural component correlated with pneumorrhachis seen on CT. A small paravertebral component extended to the left psoas and adjacent muscles, with the left psoas showing altered signal intensity and bulky appearance. Post-contrast imaging revealed peripheral enhancement and multiple loculations within the left psoas, consistent with an emphysematous abscess of approximately 50-60 cc volume (Figures 4, 5).

**Figure 6:**
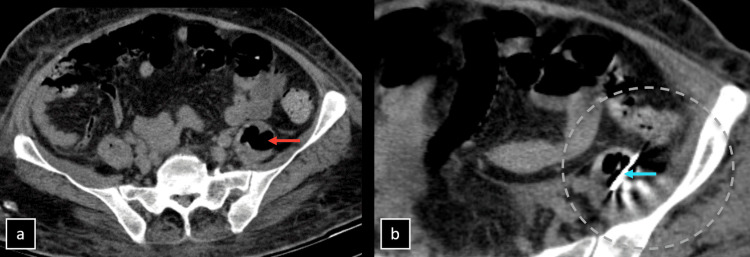
Non-contrast-enhanced computed tomography (CT). Non-contrast-enhanced CT, axial sections show a (a) pre-procedural image displaying the left psoas abscess (orange arrow) and (b) precise placement of the tip of an 18-gauge spinal needle within the left psoas abscess (cyan arrow), highlighting the technical precision of the CT-guided catheter drainage procedure.

In summary, the imaging findings indicated pathology originating in the lumbar spine, likely as a consequence of an infectious process leading to the formation of an emphysematous abscess. The interventional radiology team performed a CT-guided catheter drainage procedure for the psoas abscess, ensuring sterile conditions throughout. An 18-gauge spinal needle was employed for the drainage, resulting in the collection of approximately 17 cc of pus (as depicted in Figure 3). The collected specimen was subsequently forwarded for microbiological culture and sensitivity testing. Pre- and post-procedure images are shown in Figure 6.

**Figure 7:**
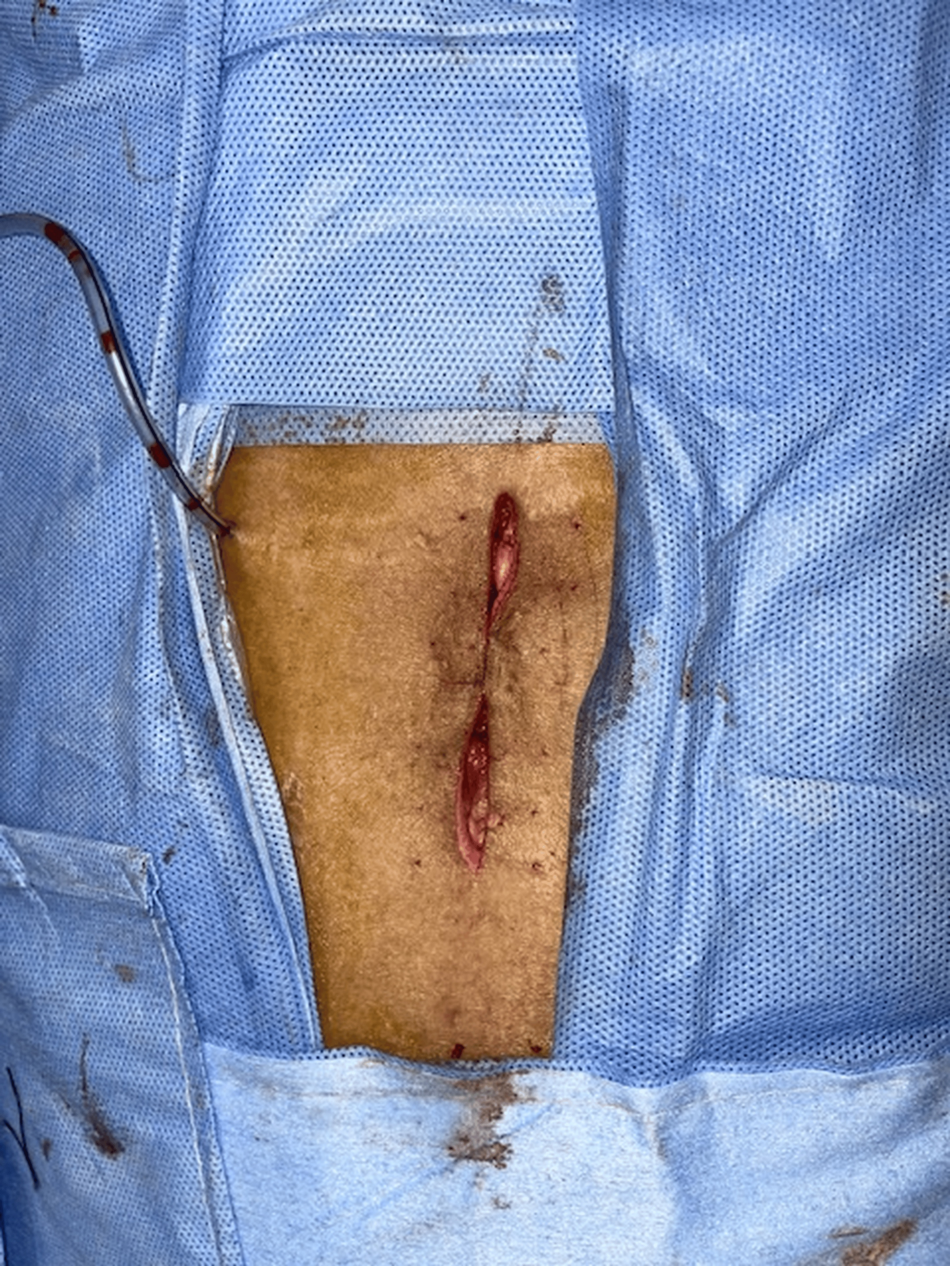
Postoperative image. Postoperative image displaying the sutured incision following an L4-L5 partial laminectomy and associated wound debridement, with a surgical drain in place.

The culture and sensitivity test for a psoas abscess revealed the presence of *E. coli*. Microscopic examination showed numerous pus cells and a few gram-negative bacilli. The organism was susceptible to a range of antibiotics, including imipenem, cefoperazone/sulbactam, piperacillin/tazobactam, cefoxitin, co-trimoxazole, and ertapenem. The patient, diagnosed with spondylodiscitis, commenced a regimen of broad-spectrum antibiotics, focusing on a combination of meropenem and piperacillin/tazobactam (Piptaz) for a four-week duration. During this period, the patient demonstrated a positive therapeutic response, as evidenced by improving laboratory values and clinical symptoms. The patient underwent a successful partial laminectomy of the L4 and L5 vertebrae, coupled with decompression of the thecal sac and meticulous debridement (Figure 7).

Clinically, the patient showed significant neurological improvement post-surgery; the muscle strength in the right lower extremity improved to 4/5, indicating good movement against resistance, while the left side exhibited a strength of 3+/5, suggesting a fair strength with the ability to overcome some resistance but not completely. This improvement in motor function was a positive indicator of recovery. After the surgery, the patient was duly discharged with instructions to attend regular follow-up appointments in the Neurosurgery Outpatient Department. These periodic reviews were essential for monitoring the patient's neurological status and wound condition, and to provide continued guidance for her rehabilitation process. Five months subsequent to the surgery, with consistent medical treatment and a dedicated rehabilitation regimen, the patient made remarkable progress. The concerted efforts of the interdisciplinary medical team and the patient's adherence to treatment and exercise protocols culminated in a return to her daily activities. She now enjoys a restored quality of life, managing her daily routines with increased mobility and independence, which stands as a testament to the success of surgical and postoperative management. 

## Discussion

EO is a rare and potentially fatal condition characterized by the presence of gas within the bone, particularly in the vertebral column [5]. Common symptoms include fever and severe pain in the affected area, such as spinal tenderness and left hip joint pain [6]. Diagnosis is typically confirmed by detecting intraosseous air on CT scans, although differential diagnosis is crucial as air density in the axial skeleton may be confused with degenerative changes, osteonecrosis, or neoplasms [1,4,5]. A diagnosis of EO is more likely if fluid collection or abscess formation is observed in adjacent tissues or if the intraosseous air exhibits an extensive and mottled pattern [3,6].

To date, there have been 49 reported cases of EO [2]. This condition affects both genders almost equally, with 25 cases in men and 24 in women [2]. The vertebral column is the most commonly affected site, accounting for 57.1% of cases, followed by the femur and pelvic bones [7]. EO frequently occurs in patients with underlying comorbidities, especially diabetes mellitus, which is present in 45% of cases [2,7,8]. In rare instances, EO can occur in individuals without known comorbidities [3,8].

The most frequent initial symptom is low back pain, reported in 65.5% of cases, often correlating with the affected area seen on imaging. Patients may also experience referred pain in the abdomen, flank, or lower extremities [2,8]. While 27.6% of patients exhibit neurological deficits, 41.4% do not [2,8,9]. Fever is present in 62.1% of cases, along with elevated WBC, CRP, and erythrocyte sedimentation rate (ESR) levels, indicating infection and inflammation [7,8]. A significant portion, 87.5%, present with sepsis, highlighting the need for early diagnostic measures, including blood and urine cultures, as well as monitoring of lactate, CRP, and ESR to assess systemic involvement [2,8,10].

The microbiology of EO predominantly involves monomicrobial infections, with *E. coli *being the most common causative organism, responsible for 37.9% of cases [10]. *Klebsiella pneumoniae* follows at 27.6%, and anaerobic bacteria, such as *Fusobacterium*, *Peptostreptococcus*, and *Clostridium*, are implicated in 26.9% of cases. The majority, 65.4%, involve members of the Enterobacteriaceae family [2,8,10]. Initial treatment typically involves broad-spectrum intravenous antibiotics, which are later tailored based on specific microbial identification. The prognosis varies, with a mortality rate of 44.4%. Among survivors, 44.4% achieve full recovery without sequelae, while 11.1% may experience neurological deficits up to six months post-infection [2,8].

Accurate diagnosis and prompt management are critical for effectively treating EO. Imaging techniques play a vital role, with CT scans being the preferred modality due to their ability to visualize gas within the bone marrow [5,9]. The characteristic "pumice stone appearance," resulting from trapped air bubbles resembling the porous texture of pumice stone, is a distinctive radiologic feature [9]. While MRI can detect early degenerative changes in the bone, CT remains the most reliable method for diagnosing EO [11].

Management of EO requires rapid diagnosis and initiation of antibiotic therapy. In some cases, surgical intervention is necessary to remove necrotic bone or address abscesses [12,13]. Surgery, performed in 37.9% of spinal EO cases, is usually reserved for cases where non-surgical treatment fails or to manage complications such as spinal cord or cauda equina compression [2,8,14]. In the reported case, treatment included psoas abscess drainage and partial laminectomy at L4 and L5 to decompress the thecal sac.

## Conclusions

This clinical encounter with a rare and severe case of spinal emphysematous osteomyelitis (EO) highlights the importance of rapid diagnostic and therapeutic intervention. Our patient, suffering from a life-threatening *Escherichia coli*-induced infection, achieved a positive outcome through swift action and targeted treatment. Despite the limited specific literature on spinal EO, this case exemplifies the potential for successful management through early recognition and appropriate therapy. It underscores the necessity for heightened awareness and prompt initiation of treatment in suspected EO cases. Accurate diagnosis, along with a combined surgical-medical approach, is crucial for achieving optimal outcomes.

By contributing to the existing knowledge base, we can refine treatment guidelines and provide clinicians with the necessary tools to manage this complex and potentially fatal condition. Continued reporting of EO cases is essential for enhancing our collective understanding and improving patient prognosis against this rare and aggressive disease.
